# Quantitative Analysis of Tremors in Welders

**DOI:** 10.3390/ijerph8051478

**Published:** 2011-05-10

**Authors:** Juan Sanchez-Ramos, Dacy Reimer, Theresa Zesiewicz, Kelly Sullivan, Paul A. Nausieda

**Affiliations:** 1 College of Medicine, University of South Florida, Tampa, FL 33612, USA; E-Mails: tzesiewi@health.usf.edu (T.Z.); kbarber@health.usf.edu (K.S.); 2 Parkinson Research Institute at Aurora Sinai Medical Center, Milwaukee, WI 53233, USA; E-Mails: dacyreimerrn@aol.com (D.R.); nausiedamd@parkcent.com (P.A.N.)

**Keywords:** manganese, toxicity, Parkinson’s Disease, tremor, accelerometer

## Abstract

**Background:**

Workers chronically exposed to manganese in welding fumes may develop an extra-pyramidal syndrome with postural and action tremors.

**Objectives:**

To determine the utility of tremor analysis in distinguishing tremors among workers exposed to welding fumes, patients with Idiopathic Parkinson’s Disease (IPD) and Essential Tremor (ET).

**Methods:**

Retrospective study of recorded tremor in subjects from academic Movement Disorders Clinics and Welders. Quantitative tremor analysis was performed and associated with clinical status.

**Results:**

Postural tremor intensity was increased in Welders and ET and was associated with visibly greater amplitude of tremor with arms extended. Mean center frequencies (Cf) of welders and patients with ET were significantly higher than the mean Cf of PD subjects. Although both the welders and the ET group exhibited a higher Cf with arms extended, welders could be distinguished from the ET subjects by a significantly lower Cf of the rest tremor than that measured in ET subjects.

**Conclusions:**

In the context of an appropriate exposure history and neurological examination, tremor analysis may be useful in the diagnosis of manganese-related extra-pyramidal manifestations.

## 1. Introduction

Reports over the last 80 years have described an increasing number of cases of manganese toxicity, especially in manganese miners, smelters, welders and battery workers (manganese dioxide) [1–5]. Although the clinical features vary in details, a general consensus appears to be that the illness (“manganism”) can present acutely with neuropsychiatric disturbances (manganese madness, or in Spanish “locura manganesa”) and more chronically with an extrapyramidal syndrome that resembles Idiopathic Parkinson’s disease (IPD) [6]. After removal from the toxic environment, psychiatric symptoms are often reversible, but the extrapyramidal signs and symptoms continue to progress [6]. Tremor has been recorded in asymptomatic miners with a history of manganese exposure, even when the last exposure was many years before instrumental tremor analysis [7].

A tremor “at rest” is considered a classic sign of IPD, while such a tremor is reported as an inconstant clinical sign in manganism. Hand tremor that worsens with activity, or with arms extended, is more typical in cases of manganism. To explore and quantify parameters of tremor in welders, a quantitative analysis of previously recorded accelerometric data was performed. The objectives of this retrospective study were (a) to characterize and analyze the tremor of welders and associated workers with a history of exposure to manganese in the workplace and (b) to compare the welders’ tremor parameters with those of *de novo* PD patients who had not been treated with anti-PD medications and to a group of patients with postural tremors, Essential Tremor (ET). The practical goal was to assess whether differences in resting and postural tremor intensities and energy distribution across the spectrum of oscillation frequency could be useful in differentiating tremor in welders diagnosed with “manganism” from IPD and ET.

## 2. Methods

### 2.1. Selection of Subjects

**Figure 1.**
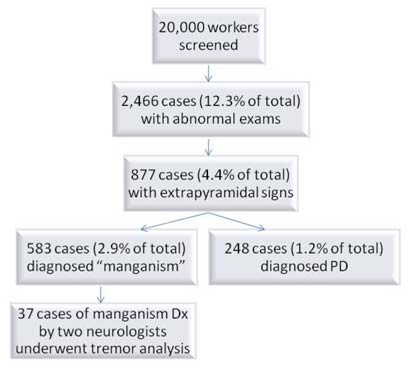
Flow chart showing selection of welders for the accelerometry study.

Approximately 20,000 individuals who worked as welders or assistants in shipyards and refineries were screened for neurologic abnormalities in a union-organized effort supported by a legal consortium over a period of five years. These subjects were self-selected by responding to advertisements that listed a number of symptoms and signs associated with manganese toxicity. A team of physicians conducted screening examinations following a simple protocol designed to detect any neurologic abnormality. The majority of the subjects who had undergone the initial screening for neurologic symptoms typically did not have extrapyramidal features, but rather exhibited signs consistent with peripheral neuropathies, radiculopathies, stroke, and occasionally demyelinating disease or motor neuron disease (see Flow Chart Figure 1). Subjects with extrapyramidal signs (slowness of movement, rigidity, tremors) were then examined in subsequent visits over three years by two neurologists (PN, JSR) with expertise in Movement Disorders. As an exploratory study, JSR recorded tremor with a portable accelerometry system (CATSYS, LTD) in a sample of 37 welders or welding assistants each of whom was examined independently on the same day (by PN and JSR) and given a consensual diagnosis of probable manganism. The syndrome of manganism was inferred from the appearance of slowness, rigidity, tremors (both resting and postural), cramping or dystonia of limbs, upper motor neuron signs, and behavioral disturbances (irritability, depression) in the context of chronic exposure to welding fumes.

Twenty “*de novo*” PD patients were used as a comparison group. These PD cases (diagnosed by PN in 2005) had undergone tremor analysis at Milwaukee Parkinson’s Disease Center of Excellence. *De novo* subjects are defined as those with recent onset of symptoms or patients whose symptoms do not yet warrant anti-PD medications. Two out of four cardinal signs were required to be present on examination (resting tremor, bradykinesia, rigidity, loss of postural reflexes) and did not have other conditions such as PD subjects who were not currently taking medication, but who had been documented to have no response to an earlier trial of Sinemet, were not included as *de novo* PD. As another comparison group, patients with mono-symptomatic postural and kinetic tremors (diagnosis of ET by TZ at USF) who had undergone tremor analysis as part of their workup were chosen in a retrospective chart review from the USF Movement Disorders Clinic.

There was no payment to individuals for their participation. Records of all cases who underwent accelerometry studies were de-identified (all personal identification was removed) prior to analysis. Review of the de-identified records of subjects who underwent accelerometric studies conducted by PN, TZ and JSR were provided an exemption by the Investigational Review Board of the University of South Florida.

### 2.2. Accelerometry

The accelerometer utilized in this study is a component of a portable-PC based test system (Catsys System) to measure coordination reaction time, tremor and postural sway or stability (Danish Product Development, Ltd) [8]. Subjects were asked to sit with their hands comfortably resting on their lap (proximal thighs) with one hand holding a pen-like stylus that contains a biaxial micro- accelerometer. The pen was held with the first three digits of the hand in a writing posture, but with the hand slightly supinated so the long axis of the pen was parallel to the ground. For the postural tremor the pen was held in a writing posture but the arms were extended anteriorly, level with the shoulders. The pen was held with the long axis parallel to the ground. The hand vibrations were recorded and displayed in real-time against a time axis plot on the computer screen. Fast Fourier Transform (FFT) analysis was used to determine the normalized power distribution of the tremor in the frequency band 0.9 Hz to 15.0 Hz. Measures derived from acceleration data were based on the Fourier power.

The tremor parameters calculated included (a) *Tremor Intensity* = root mean square of accelerations expressed as m/s^2^ recorded in the 0.9–15 Hz band; (b) *Center frequency (Cf)* = mean frequency of accelerations in the 0.9 Hz to 15.0 Hz band; (c) *Frequency Dispersion* = degree of irregularity of the tremor defined as the frequency band centered around the median frequency containing 68% of the power; (d) *Energy distribution*= relative energy distributed in different frequency band (3–56.5 Hz and 6.6–10 Hz; (e) *Harmonic Index* = tremor frequency pattern with the pattern of a single harmonic oscillation, which has a HI = 1.00. HI decreases when tremor is composed of many oscillations.

### 2.3. Statistical Analysis

Group data was expressed as mean and standard error of the mean (SEM). After ascertaining the distribution of data was Gaussian, two-way analysis of variance (ANOVA) was followed by t-tests when appropriate. Fisher’s Exact Test was used to analyze contingency table data. All statistical analyses, including linear regression analyses relating age or duration of symptoms to various tremor parameters, was performed using Software from Graphpad Prism, Inc. (San Diego, CA).

## 3. Results and Discussion

Comparison of the mean ages of three groups at the time of this study revealed that the welders were younger (mean of 54.8 years) than both the IPD patients (mean of 65.3 years) and the ET subjects (mean of 59 years). See Table 1. The proportion of males among the three groups also differed. Extrapyramidal symptoms began at an earlier age in the welders (47.9 years) and ET subjects (50 years) compared to the IPD patients (59.5 years). The welders reported an average of 25.6 ± 10.8 years of exposure to welding fumes. The time since the last exposure to fumes was 7.8 ± 10.7 years. None of the subjects had been treated with anti-parkinson medications.

The initial symptoms reported by the subjects did not differ significantly between welders and IPD subjects. Tremor, slowness, balance difficulties and cramping of the limbs were reported with equal frequency in each group (See Table 2). All of the ET patients presented with tremors. The clinical examination revealed significant differences in the characteristics and distribution of tremors. A greater proportion of welders exhibited action and postural tremors than the IPD patients. All of the ET patients exhibited postural tremors. Tremor, bradykinesia and rigidity were significantly more asymmetrical in the IPD patients than in the welders. Interestingly, the frequency of cog-wheel rigidity and dystonic limb posture in welders was not significantly different from the IPD group. Dyshydrosis, or excessive night sweats, was significantly more common in the welders.

### 

#### Quantitative Tremor Analysis

The clinical observation that tremor was worsened with arms extended in welders and ET patients, but not in in IPD patients was substantiated by quantitative tremor analysis. In addition, the center frequency (Cf) of oscillation of the postural tremors was significantly higher in the welders and the ET group than in IPD. The mean harmonic indices for both resting and postural tremors ranged from 0.91 to 0.94 in all three groups, indicating that the tremors in both conditions tend to oscillate around a single frequency, a feature typical for pathological tremor (group data is shown in Tables 3, 4, 5).

In the welders, the mean intensity of tremor increased more than 2-fold when the right arm was extended (from 0.774 to 1.61 m/s^2^); with the left arm the intensity increased by 1.51 fold (from 0.706 to 1.07 m/s^2^). In the PD group, the intensity of tremor did not change significantly with arms extended. In the ET group, the intensity of tremor increased 2.4 fold on the right (from 0.24 to 0.58) and 4.8 fold on the left (from 0.12 to 0.59) with arms extended.

**Figure 2.**
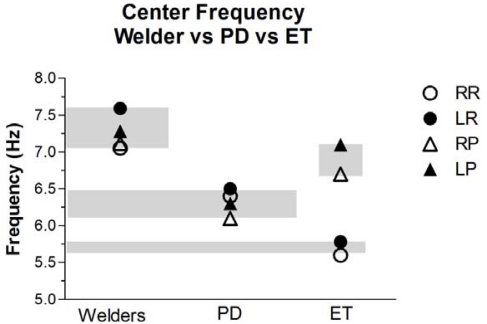
Center Frequency (Cf) Analysis. The mean Cf for the tremors are significantly higher in Welders compared to PD under all conditions (right *vs*. left) and (resting *vs.* postural); paired two-tailed t-test (p = 0.009). The mean Cf of the *postural* tremors are higher in the welders compared patients with ET, but does not reach statistical significance. However, the Cf of the *rest* tremors in welders are significantly higher than in patients with ET. 2-way ANOVA of Cf of the ET group showed that rest *vs.* posture, but not right *vs*. left, contributed significantly to total variance (p = 0.009). The grey bands indicate the range of the mean Cf for each group of subjects and are intended to facilitate visual comparisons.

The mean Center Frequency (Cf) of the postural tremors was significantly higher in welders and ET group compared to PD subjects (Figure 2). However, the Cf of the rest tremor in the ET group was significantly lower than the Cf of the rest tremor of the welders. The higher Cf in welders and ET compared to PD subjects was confirmed by analysis of energy distribution into two frequency bands, 3–6.5 Hz and 6.6 to 10 Hz. The resting tremor energy distribution in welders revealed a greater relative intensity at the higher frequency band (43% of total energy in both right and left hands) compared to the lower frequency band (27% on the left hand and 35% on the right). (Data not shown). In contrast, analysis of the PD subjects revealed that 53% of the total energy was distributed in the lower frequency band and 25% was in the higher frequency band (6.6 to 10 Hz). Analysis of the postural data revealed the same distribution pattern. In the ET group, a greater percentage of the energy of *resting tremor* was found in the lower frequency band (3 to 6.5 Hz), but with arms extended, the energy was equally distributed across both high and low frequency bands.

Clinically, the higher Cf in welders and ET patients corresponds to a more rapid oscillation of the hands with arms extended than that typically seen in IPD subjects. Although both the welders and the ET group exhibited a higher Cf with arms extended, welders could be distinguished from the ET subjects by a significantly slower Cf of the rest tremor than that measured in ET subjects.

Asymmetry of tremor intensity was observed in individual patients within the three groups and there was clear asymmetry in the clinical evaluations of IPD patients. The group analysis of tremor intensity data did not reveal statistically significant left/right differences in IPD and ET because the asymmetries in these cohorts were just as likely to be L > R as R > L. However, significant side-to-side group difference in the tremor intensity (R > L) was observed in postural tremors of the welders cohort.

**Figure 3.**
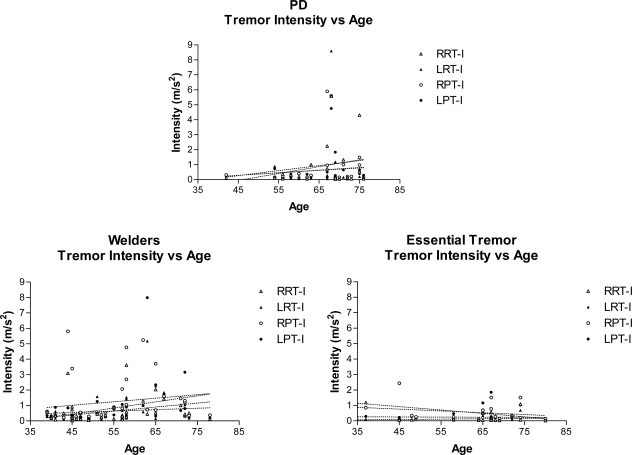
Tremor Intensity *vs.* Age of Subjects. Tremor intensity (m/s^2^) of right and left hands, measured both at rest and with arms extended (postural tremor), does not change significantly with age of welders, PD subjects or essential tremor. Linear regression analysis revealed that the slopes of the lines did not differ significantly from zero for resting and postural tremors measured in each limb.

**Figure 4.**
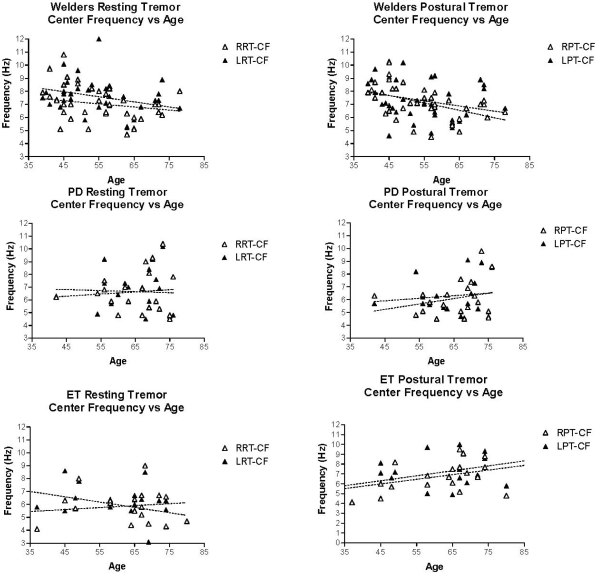
Center Frequency *vs.* Age. Upper panels show the results of linear regression analysis in welders. The slope of the lines does not differ significantly from zero with the exception of the right sided postural tremor in which Center frequencies show an age-dependent decrease (p < 0.05). The middle panels show the results of linear regression analysis in PD subjects. None of the slopes differ significantly from zero. The lower panels (ET) indicate an age-dependent increase in postural tremor Cf on the right side. Regression line is significantly different from zero (p = 0.04).

**Figure 5.**
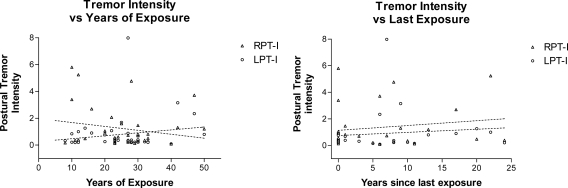
Relationship between tremor intensity and duration of exposure or years since last exposure to welding fumes. Linear regression analysis of intensity of postural tremor against years of exposure (left panel) and years since last exposure (right panel) revealed no significant relationship. Slope was not significantly different from zero in any of the analyses.

Regression analysis was performed on tremor intensity, and Cf as a function of age in the three groups of patients. There was no significant change in tremor intensity with age in any of the three groups (Figure 3). Linear regression analysis also showed no significant relationship between Cf and age in PD subjects. However, welders showed an age-dependent decrease in the Cf of right sided postural tremor while patients with ET showed an age-dependent increase in Cf (p = 0.04). (Figure 4) Linear regression analysis of tremor intensity (and other parameters of tremor) in welders against duration of symptoms or time last exposed to welding did not find significant relationships (Figure 5).

**Figure 6.**
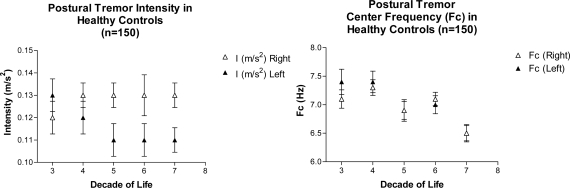
Tremor Parameters in Healthy Controls. Left panel shows postural tremor intensity as a function of decade of life. Right panel shows Center frequencies of postural tremor against decade of life. Data are derived from accelerometer recordings of postural tremor using the Catsys Accelerometer system in 150 healthy controls (75 females and 75 males). Data were plotted from a published table of normal values [8].

Comparing tremor parameters of welders, IPD and ET subjects with published norms of healthy controls is instructive. In both the welders and the IPD group, the tremor intensity was significantly greater than in healthy controls measured with the same Catsys system. The mean intensity of postural tremor hovers between 0.11 and 0.13 throughout five decades of life (Figure 6). In contrast the postural tremor intensities of welders averaged 1.07 on the left and 1.61 on the right (a 12.4- and 9.7-fold increase, respectively, from healthy controls). Postural tremor intensities in the IPD subjects were also significantly elevated, up to 7.5-fold of healthy controls. In the ET group, the Cf increased with age. Interestingly, the Cf of postural tremor in controls declines normally with age, starting around 7.25 Hz in the 3rd and 4th decade of life and approximating 6.5 Hz by the 7th decade.

Previous work on the quantitative analysis of fine motor function of subjects exposed to low-levels of manganese has documented an association between manganese exposure and a decrease in ability to perform regular, rapid and precise pointing movements, and a decrease in ability to attain high maximum rotation speeds in rapid alternating movements. Using the same Catsys accelerometry system, researchers also found an increase in regularity of tremor oscillations, indicated by a narrow dispersion around center frequency [9].

More recently, researchers from Quebec studied 10 workers previously exposed to manganese, 10 patients with PD and 11 control subjects, employing a transducing laser technique to quantify postural tremor [10]. The tremor of workers with a documented exposure to Mn could be adequately described with only two variables: the “corrected wobble”, which describes the morphology of the tremor oscillations, and the variability ratio which describes the proportional power of tremor. The proportion of the power spectrum in the 7–12 Hz range was an important discriminating variable in distinguishing subjects exposed to manganese from patients with PD just as it was in the results described here. Interestingly, blood Mn levels in workers were normal at the time of the experiment and yet the effect of prior exposure to Mn on postural tremor was still detected by this sensitive method [10].

Tremor has been recorded in asymptomatic subjects with a history of manganese exposure, even when the last exposure was many years before the examination. Tests of resting and postural tremor and hand coordination have documented abnormalities in asymptomatic miners with an average of 20 years exposure to manganese dust in Chilean manganese mines [7]. A computerized protocol based on data acquired with a digital tablet was utilized to measure tremor and coordination in that study. Since it was not based on accelerometery, their tremor parameters cannot be directly compared to the present findings based on Fast Fourier transformation of measured oscillations. However, it should be pointed out that sub-clinical abnormalities in tremor and coordination were found at least 5 years after the last exposure. The authors of that study concluded that chronic asymptomatic manganese exposure may result in detectable late-life abnormalities of movement.

The present study suffers from several shortcomings including selection bias, examination of the ET and IPD groups by different examiners and inadequate documentation of intensity of exposure to welding fumes. The diagnosis of manganism among the 20,000 self-referred welders yielded 583 cases (chosen on the basis of neurological findings in the context of a long exposure history), but the 37 cases that underwent accelerometric recordings were not randomly selected and hence may not represent the larger population of affected welders. Estimates of the prevalence of parkinsonism among welders has been reported to be as high as 1.36% of the population of welders in Alabama (1,366/100,000) [11], and the percentage of cases of manganism among the self-referred welders in this study was even higher (2.9%). It is not possible or appropriate to compare results from an epidemiological study of parkinsonism in welders with the present report, which was not designed to estimate frequency of manganism but rather to focus on quantitative tremor analysis in a small sample of welders. Another problem is that the cohort of welders selected was based on the diagnosis of manganism by two independent movement disorders specialists while the other two cohorts (ET and IPD) were selected on the basis of a single neurologist’s diagnosis. It is possible that different raters examining the different study groups may increase uncertainty in distinguishing differences among the diagnostic groups, but at the very least, the criteria for diagnoses were clearly defined. Another limitation of this study might be an inadequate documentation of manganese exposure, relying only on historical data. Documentation of exposure by serum or urinary levels of Mn and neuroimaging would of course be important in the description of acute manganism. However, as discussed above, quantitative abnormalities of tremor can be found in chronically exposed asymptomatic subjects and in workers with normal serum Mn levels [7,10].

## 4. Conclusions

Postural tremor intensity was increased in Welders and ET and was associated with visibly greater amplitude of tremor with arms extended. Mean center frequencies (Cf) of welders and patients with ET were significantly higher than the mean Cf of PD subjects. Although both the welders and the ET group exhibited a higher Cf with arms extended, welders could be distinguished from the ET subjects by a significantly lower Cf of the rest tremor than that measured in ET subjects. In the context of an appropriate exposure history and neurological examination, tremor analysis may be useful in the diagnosis of manganese-related extra-pyramidal manifestations.

It is important to emphasize that tremor analysis in isolation is not sufficient to implicate manganese exposure as the cause of the abnormal oscillations. The results presented here demonstrate that quantitative tremor analysis can provide additional objective information to the clinical evaluation of patients with possible manganese-induced parkinsonism. Validation of these findings will require prospective studies in which quantitative tremor analysis is performed separately by a blinded investigator without knowledge of clinical diagnosis. The sensitivity and specificity of tremor analysis for predicting the correct diagnosis can then be determined. Quantitative tremor analysis may be useful in the diagnosis of individual cases of manganese-induced parkinsonism to the extent that there is an appropriate documentation of exposure and a careful neurological examination.

## Figures and Tables

**Table 1.** Age at onset of symptoms.

	**Welders (n = 37)**	**IPD (n = 20)**	**ET (n = 20)**

**Males/Female**	36/1	13/7	9/11
**Age**	54.8 ± 10.7	65.3 ± 9.15	59.5 ± 14.9
**Age at Onset**	47.9 ± 11.7	59.5 ± 9.68	50 ± 14.8 (n = 12)
**Years of Exposure to Welding Fumes**	25.6 ± 10.8	—	—
**Years since last Exposure**	7.8 ± 10.7	—	—

**Table 2.** Symptoms and signs.

	**Welders**	**IPD**	

	*Number of subjects with sign/total examined (%)*		*Significance level*

**Initial symptoms (based on history)**			
Tremor	29/32 (90.1%)	14/17 (82%)	p = 0.41
Bradykinesia	5/32 (15.6 %)	3/17 (17.6%)	p = 1.0
Balance	7/31 (22.5%)	1/17 (5.8%)	p = 0.23
Limb cramping	7/32 (21.8 %)	0/17 (0%)	p = 0.08

Current signs (based on UPDRS) *			
Resting tremor (Item 20 >0)	19/31 (61.2%)	9/16 (56.2%)	p = 0.76
Action tremor (Item 21>0)	27/31 (87%)	3/16 (18.7%)	* p < 0.0001
Postural tremor (Item 21≥3)	21/31 (67.7%)	2/16 (12.5%)	* p < 0.0005
Dystonic limb posture	1/31 (3.2%)	1/17 (5.8%)	p = 0.66
Cog-wheel rigidity (Item 22>0)	8/29 (27.5%)	3/17 (17.6%)	p = 0.45

Distribution of signs (based on UPDRS) *			
Asymmetric Tremor UE (Item 20 R≠L)	4/29 (13.7%)	10/16 (62.5%)	* p = 0.002
Asymmetric Rigidity UE (Item 22 R≠L)	11/29 (37.9%)	11/12 (91.6%)	* p = 0.002
Asymmetric Brady UE (Item 23 R≠L)	11/29 (37.9%)	11/11 (100%)	* p = 0.003

**Autonomic nervous system (based on history)**			
Dyshidrosis	11/29 (37.9%)	0/17 (0%)	* p = 0.01
Dizziness on standing	0/29 (0%)	1/17 (5.8%)	p = 0.25

* Current signs and distribution of signs were assessed using the Unified Parkinson’s Disease Rating Scale (UPDRS-Part III). Item 20 (tremor at rest); Item 21 (action or postural tremor of hands; Item 22 (Rigidity); Item 23 (Finger tap speed). Fisher’s Exact Test was used to analyze contingency table data.

**Table 3.** Welders tremor analysis.

	**Welders (n = 37)**							
	**Resting Tremor**				**Postural Tremor**			
	*Intensity (m/s^2^)*	*Center Freq. (Hz)*	*Center Freq. Dispersion (Hz)*	*Harmonic Index*	*Intensity (m/s^2^)*	*Center Freq. (Hz)*	*Center Freq. Dispersion (Hz)*	*Harmonic Index*
**Right side** Mean ± SEM	0.774 ± 0.194	7.05 ± 0.263	1.19 ± 0.160	0.941 ± 0.041	1.61 ** ± 0.457	7.12 ± 0.208	1.36 ± 0.204	0.910 ± 0.066
**Left side** Mean ± SEM	0.706 ± 0.175	7.59 ± 0.227	1.90 ± 0.187	0.917 ± 0.049	1.07 ± 0.335	7.28 ± 1.54	2.04 ± 0.707	0.936 ± 0.055

Two way ANOVA revealed that arm position (resting *vs.* postural) accounted for 2.48% of variance (p = 0.05); right-left accounted for 0.64% of total variance (p = 0.33); the interaction of right-left with position accounted for 0.38% of the variance (p = 0.45).

** Paired t-test comparing Welders resting tremor to postural tremor intensity revealed a significant increase with right arm extended (p < 0.006) and a trend towards an increase with left arm extended (p = 0.07).

**Table 4.** IPD tremor analysis.

	**PD (n = 20)**							
	**Resting Tremor**				**Postural Tremor**			
	*Intensity (m/s^2^)*	*Center Freq. (Hz)*	*Center Freq. Dispersion (Hz)*	*Harmonic Index*	*Intensity (m/s^2^)*	*Center Freq. (Hz)*	*Center Freq. Dispersion (Hz)*	*Harmonic Index*
**Right side** Mean ± SEM	0.867 ± 0.338	6.64 ± 0.367	1.93 ± 0.336	0.946 ± 0.045	0.937 ± 0.377	6.10 ± 0.315	2.21 ± 0.363	0.94 ± 0.058
**Left side** Mean ± SEM	0.633 ± 0.424	6.65 ± 0.377	1.59 ± 0.346	0.944 ± 0.038	0.613 ± 0.235	6.32 ± 0.323	2.24 ± 0.299	0.936 ± 0.050

Two way ANOVA revealed that neither arm position (resting *vs.* postural) nor right *vs.* left had a significant effect on the tremor intensity.

**Table 5.** Essential tremor analysis.

	**ET (n = 20)**							
	**Resting Tremor**				**Postural Tremor**			
	*Intensity (m/s^2^)*	*Center Freq. (Hz)*	*Center Freq. Dispersion (Hz)*	*Harmonic Index*	*Intensity (m/s^2^)*	*Center Freq. (Hz)*	*Center Freq. Dispersion (Hz)*	*Harmonic Index*
**Right side** Mean ± SEM	0.241 ± 0.076	5.829 * ± 0.291	1.755 ± 0.272	0.956 ± 0.007	0.588 ** ± 0.140	6.674 ± 0.340	2.580 ± 0.306	0.936 ± 0.008
**Left side** Mean ± SEM	0.124 ± 0.035	5.995 * ± 0.355	2.270 ± 0.330	0.939 ± 0.008	0.599 ** ± 0.175	7.055 ± 0.386	2.855 ± 0.305	0.918 ± 0.010

** Postural tremor intensities were significantly greater than resting tremor intensity (p < 0.05).

* Rest *vs*. Postural Cf contributes significantly to total variance (p = 0.009).
